# A Transcript Cleavage Factor of *Mycobacterium tuberculosis* Important for Its Survival

**DOI:** 10.1371/journal.pone.0021941

**Published:** 2011-07-08

**Authors:** Arnab China, Sonakshi Mishra, Valakunja Nagaraja

**Affiliations:** 1 Department of Microbiology and Cell Biology, Indian Institute of Science, Bangalore, India; 2 Jawaharlal Nehru Centre for Advanced Scientific Research, Bangalore, India; Queen Mary University of London, United Kingdom

## Abstract

After initiation of transcription, a number of proteins participate during elongation and termination modifying the properties of the RNA polymerase (RNAP). Gre factors are one such group conserved across bacteria. They regulate transcription by projecting their N-terminal coiled-coil domain into the active center of RNAP through the secondary channel and stimulating hydrolysis of the newly synthesized RNA in backtracked elongation complexes. *Rv1080c* is a putative *gre* factor (*Mtb*Gre) in the genome of *Mycobacterium tuberculosis*. The protein enhanced the efficiency of promoter clearance by lowering abortive transcription and also rescued arrested and paused elongation complexes on the GC rich mycobacterial template. Although *Mtb*Gre is similar in domain organization and shares key residues for catalysis and RNAP interaction with the Gre factors of *Escherichia coli*, it could not complement an *E. coli gre* deficient strain. Moreover, *Mtb*Gre failed to rescue *E. coli* RNAP stalled elongation complexes, indicating the importance of specific protein-protein interactions for transcript cleavage. Decrease in the level of *Mtb*Gre reduced the bacterial survival by several fold indicating its essential role in mycobacteria. Another Gre homolog, Rv3788 was not functional in transcript cleavage activity indicating that a single Gre is sufficient for efficient transcription of the *M. tuberculosis* genome.

## Introduction

Once the process of transcription is initiated by RNAP, it is important for the enzyme to carry out elongation and termination to ensure the full-length RNA synthesis. However, the movement of the RNAP along the template during the transcription elongation is not uniform and gets interrupted either accidentally or due to regulatory mechanisms [1]. Inadvertent disruption of the elongation complex would lead to the accumulation of non-functional RNA which can be potentially deleterious to the cell [2]. To overcome these interruptions, a number of transcription factors act during elongation and termination by modifying the properties of RNAP [1], [3], [4]. These factors deal with the accidental disruption of the elongation process and affect transcription processivity and fidelity by modulating pausing, arrest, termination or anti-termination of the enzyme [1], [5]. Prokaryotic transcript cleavage factors GreA and GreB [6], [7] and their eukaryotic analog, elongation factor TFIIS [8], stimulate intrinsic transcript cleavage activity of RNAP [9], [10] for removal of the aberrant RNA 3′ ends so that polymerization activity can be restored from the end of a cleaved RNA. They suppress the RNAP pausing to rescue arrested [7], [11] or road-blocked [12] transcription complexes, providing RNAP a second chance to resume elongation [13] by directly accessing the RNAP active center through the secondary channel [10], [14]. Although homologs of the Gre factors are found in most bacteria, they are well characterized only from a few species *viz. E. coli* [6], [7], *Thermus thermophilus* and *Thermus aquaticus* [15], [16], [17]. No information on the properties of the transcript cleavage factors is available from genus mycobacteria which harbors several pathogenic species. In this manuscript we describe the characteristics of *M. tuberculosis* Gre factor.

The genome of *M. tuberculosis* harbors a *gre* factor - *Rv1080c* [18], sharing 32% and 26% identity (48% and 43% similarity) with the *E. coli* GreA and GreB respectively. Other ORFs which show low degree of similarity with the *E. coli* Gre factors in the genome are *Rv3788* which shares 16% identity and 33% similarity with the *E. coli* GreA (Figure S1A) and *Rv2103* – a hypothetical protein, having much lower similarity (9% identity and 21% similarity with *E. coli* GreA). The former has Gre like domain organization while the latter lacks key acidic amino acids and the domains required for Gre like activity.

A number of molecular processes show significant differences in mycobacteria compared to the other well-studied bacterial systems [19]. Presence of a large number of sigma factors recognizing unique sequences of the promoters in their GC rich genomes [20], slow rates of transcription and macromolecular synthesis [21], [22] and occurrence of novel transcription activators [18] etc. point towards the differences in the transcription process. The GC rich genome of *M. tuberculosis* (65.6% G+C) may pose additional challenges to the transcribing RNAP and hence the role of Gre factor could be critical for high fidelity transcription. We demonstrate that *Rv1080c*, the primary Gre factor of the genome is essential for cell survival unlike the Gre factors characterized from other eubacteria. The protein is needed for efficient promoter escape by reducing the abortive initiation and anti-arrest action during transcription elongation. Although its properties resemble *E. coli* GreA in many respects, it does not appear to collaborate with *E. coli* RNAP during elongation process and much of its properties seem to be tailored for the mycobacterial transcription.

## Results

### *Rv1080c* has Gre factor like domain organization

*Rv1080c* encodes for a 164 amino-acid protein having sequence similarity with the *E. coli* transcript cleavage factors GreA and GreB (Figure S1). A homology model of the protein was generated by using the crystal structure of *E. coli* GreA (PDB code:1GRJ) [23] as a template (Figure S1B). GreA and GreB of *E. coli* have two distinct domains: an N-terminal coiled-coil (Gre-NTD) and a C-terminal globular domain (Gre-CTD) [14], [24], [25]. NTD is responsible for the stimulation of specific nucleolytic and anti-arrest activities, whereas the residues in Gre-CTD interact with RNAP-β′ subunit coiled-coil domain [26], [27]. From the model, it is evident that Rv1080c is more similar to the *E. coli* GreA than GreB in its surface charge distribution (Figure S1C). The homology model of Rv3788, the other Gre homolog in the *M. tuberculosis* genome, shows that most of the features of the Gre factor are conserved in the ORF (Figure S1A, S1B and S1C). The *M. smegmatis* Gre (*Ms*Gre) has 97% similarity with the *M. tuberculosis* protein in the amino acid sequence and shares similar domain architecture. To understand the function and the nature of transcript cleavage stimulatory activity of mycobacterial Gre factor and the Gre factor homolog Rv3788, the genes were cloned in pET20b for over-expression of the ∼18 kDa proteins in *E. coli* (Figure S2A, S2B and S2C). The identities of the expressed proteins were confirmed by peptide-mass-fingerprinting using MALDI-TOF (data not shown).

### *Mtb*Gre stimulates the intrinsic cleavage activity of mycobacterial RNAP

**Figure 1:**
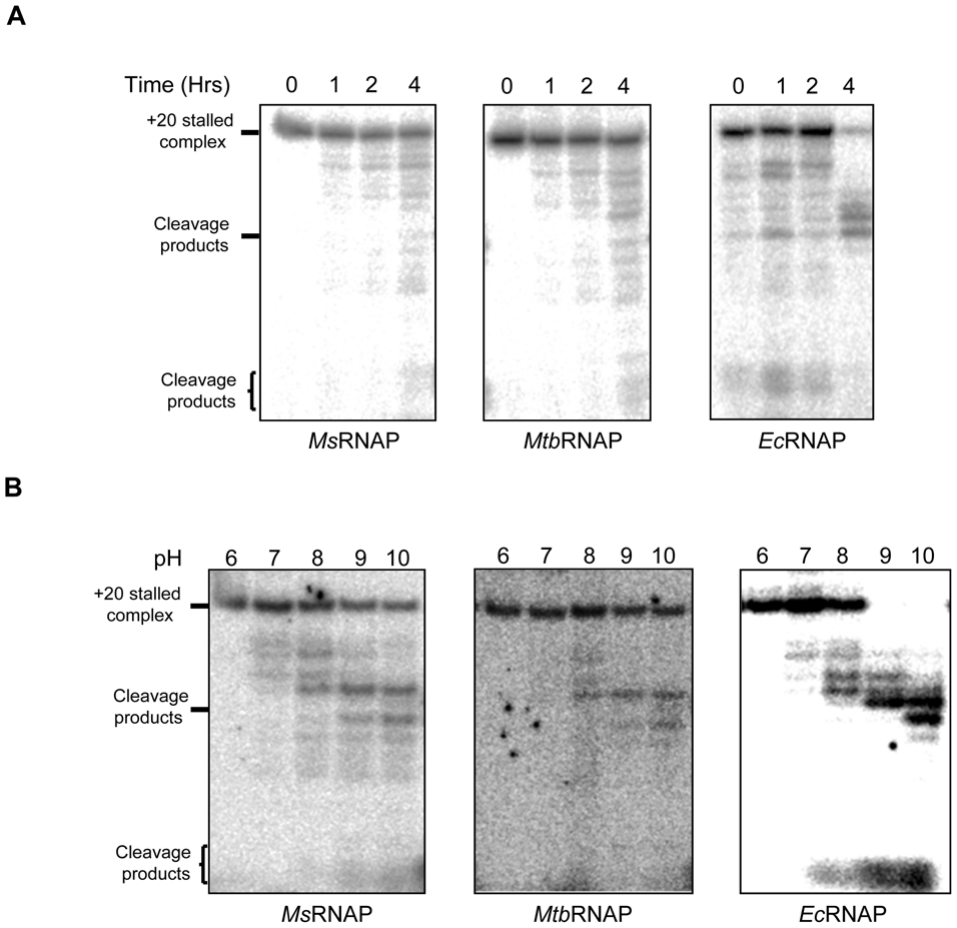
(A) Intrinsic transcript cleavage property of RNAP. Stalled elongation complexes bearing the 20 mer transcript were generated with *M. smegmatis* (*Ms*), *M. tuberculosis* (*Mtb*) and *E. coli* (*Ec*) RNAP respectively. The complexes were incubated for a prolonged time (1–4 hrs) in transcription buffer (pH 7.5), followed by resolving the cleavage products on 20% urea-PAGE. **(B)** pH-induced transcript-cleavage activity of RNAP. The gels show cleaved RNA generated from the 20 mer ternary complexes formed by *Ms*, *Mtb* and *Ec* RNAP in buffers of pH 6.0 to 10.0.

A stalled elongation complex comprising of 20 nt RNA was generated from the T7A1 promoter (T7A1-TEC) for studying transcript cleavage on the elongation complexes (Figure S3A). RNAP from both *M. smegmatis* (*Ms*RNAP) and *M. tuberculosis* (*Mtb*RNAP) were proficient in carrying out transcription from this template (Figure S3B). Transcript cleavage is an intrinsic property of the catalytic center of the RNAP [9] but is very slow and requires prolonged incubation. First, this intrinsic cleavage activity of the enzymes from *E. coli*, *M. smegmatis* and *M. tuberculosis* were compared. In all the three enzyme systems, RNA fragments of varied length were generated after incubation for a few hrs. Varied amount of short RNA fragments generated from the 3′ end of the stalled TEC could be detected at the bottom of the gels (Figure 1A). Both *Mtb*RNAP and *Ms*RNAP had lower intrinsic cleavage compared to *E. coli* RNAP (*Ec*RNAP) (Figure 1A), but the cleavage activity was stimulated in alkaline pH similar to the *E. coli* enzyme (Figure 1B) indicating the conservation of the mechanism across different bacterial species. However, the cleavage of the TEC was not complete for the mycobacterial RNAPs even at alkaline pH. The slower nuclease activity seen above was inherent to the mycobacterial polymerases and not due to the co-purification of endogenous Gre factor (Figure S4).

**Figure 2:**
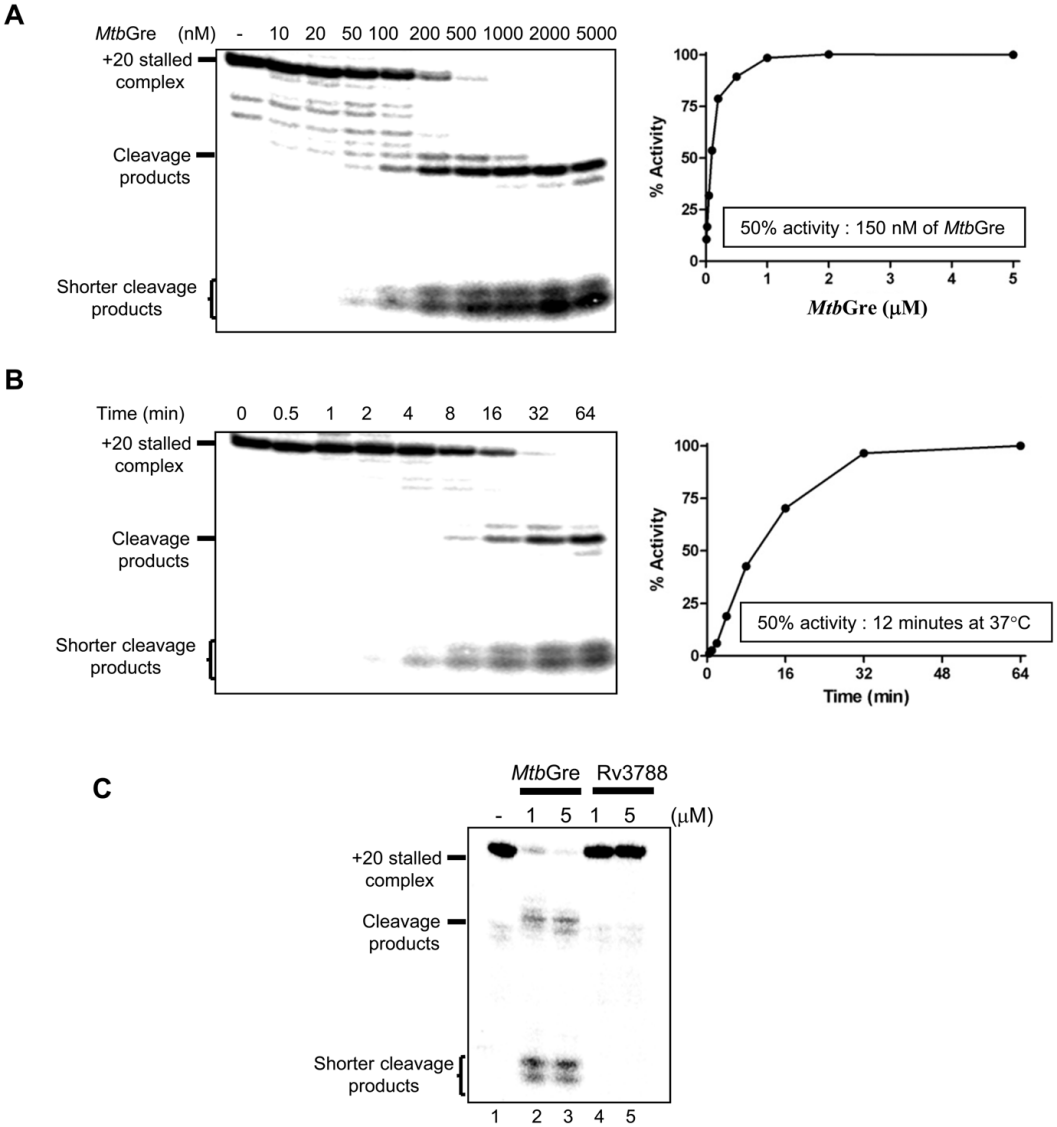
*Mtb*Gre factor stimulates the cleavage of 20 mer transcript. (**A**) Determination of the unit activity of *Mtb*Gre. Stalled TEC generated with *M. smegmatis* RNAP was incubated with different concentrations of *Mtb*Gre (10 nM to 5 µM) for 30 mins. Reactions were terminated and resolved on a 20% urea PAGE. (**B**) Time-course of *Mtb*Gre activity. Stalled TECs were incubated at 37°C with 1 µM *Mtb*Gre and aliquots were removed at different time points and quenched with urea gel loading dye followed by resolving on a 20% urea PAGE. The time required for 50% cleavage of the TEC was calculated from the plot. (**C**) The *Mtb*Gre homolog-Rv3788 does not induce transcript cleavage. *Mtb*Gre could induce the cleavage of +20 nt stalled elongation complex at T7A1 template (lanes 2 and 3). Rv3788 does not have detectable transcript cleavage stimulatory activity (lanes 4 and 5).

*Mtb*Gre factor stimulated the cleavage of short fragments (2–3 nt) from the 3′ end of the nascent RNA in 20-mer T7A1-TEC, and 50% of the cleavage could be achieved in less than 12 minutes (Figure 2A and 2B) indicating that *Rv1080c* indeed functions like a Gre factor. The pattern seen with *Ms*Gre was nearly identical mirroring their high degree of similarity (Figure S5A). However, its transcript cleavage activity appears to be higher compared to the *Mtb*Gre. In *E. coli*, GreA - induced hydrolysis generates mostly shorter di- and tri-nucleotides (type I cleavage), while GreB - induced hydrolysis generates variable length of fragments up to 18 nt in length (type II cleavage) depending on the extent of RNAP backtracking [6], [7], [28]. The pattern shown in Figure 2A and 2B and Figure S5A indicate that mycobacterial Gre factor follows type I cleavage.

The *Mtb*Gre homolog – Rv3788 is a protein of 161 amino acids with a predicted coiled coil N-terminal domain and C-terminal globular domain (Figure S1A and S1B). The key acidic residues required for transcript cleavage activity of Gre factors and the hydrophobic residues in the C-terminal RNAP interaction region are conserved in Rv3788. However, the transcript cleavage assays presented in Figure 2C show that Rv3788 lacks the cleavage stimulatory activity on the stalled elongation complexes in assay conditions used for canonical Gre factor and hence not investigated further.

### Gre factor knock-down results in growth retardation in mycobacteria

**Figure 3:**
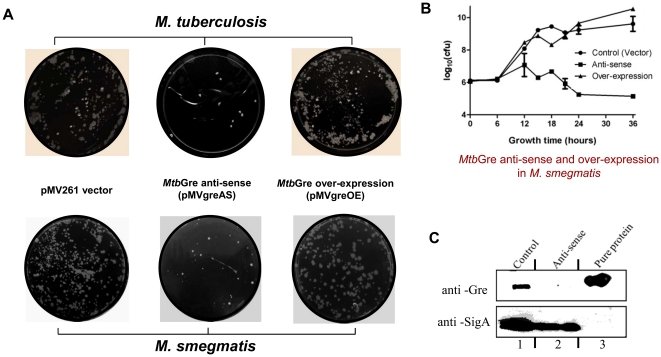
Knock-down of *gre* in mycobacteria is deleterious to the cell growth. (**A**) The pMV261-vector control, *Mtb*Gre anti-sense (pMV*greAS*) and *Mtb*Gre over-expressing (pMV*greOE*) *M. smegmatis* and *M. tuberculosis H37Ra* cells were grown for 18 hrs or 7 days respectively in liquid cultures at 37°C under shaking condition, serially diluted (10^−7^ for vector control and over-expression and 10^−5^ for anti-sense) and plated to determine the cell viability. (**B**) Growth curve of *M. smegmatis* cells with vector control, *Mtb*Gre anti-sense and *Mtb*Gre over-expression constructs. Cultures were diluted in Middlebrook 7H9 broth to give an initial OD _600_ of 0.02 to 0.04 and incubated for 36 hours. The growth curves were plotted by measuring cell viability by dilution plating at different time points. (**C**) Western blot of the *M. smegmatis* cell lysates from vector control (lane 1) and anti-sense *Mtb*Gre construct (lane 2) using a polyclonal antibody against *Mtb*Gre. Purified *Mtb*Gre has been used as a positive control (lane 3).

To check the importance of *gre* factor for cell growth, an anti-sense construct was generated by cloning the *M. tuberculosis gre* gene in reverse orientation under the control of the constitutive *hsp60* promoter in pMV261 (Figure S5B). This strategy has been successfully employed to assess the physiological importance of several other mycobacterial genes [29], [30], [31]. The expression of *M. tuberculosis gre* anti-sense reduced the viability *M. tuberculosis* (Figure 3A) by several folds compared to the control cells transformed with only pMV261 vector. *M. smegmatis* cells transformed with the *Mtb*Gre anti-sense construct also showed reduced viability (Figure 3A) and were compromised in growth when compared to the cells transformed with vector or *Mtb*Gre over-expressing construct (Figure 3B). Western blots of the cell lysates probed with anti-Gre antibody showed highly reduced level of Gre protein in the cells with anti-sense construct, suggesting that the decreased survival could be due to the reduction in Gre concentration in the cells (Figure 3C). The *M. smegmatis* cells over-expressing *Mtb*Gre factor also showed an elongated phenotype (Figure S5C).

From the above data, it is apparent that the decrease in intracellular Gre levels could have caused the growth defects in both the organisms. This would also mean that a balanced pool of Gre may be required to sustain the cell viability. To measure the endogenous levels of the protein, semi-quantitative western blot analysis was carried out at different stages of cell growth. The expression level of the endogenous Gre was highest in mid-exponential phase, both in *M. smegmatis* and in *M. tuberculosis* (Figure S6A). The Gre concentration in *M. smegmatis* was ∼82 fmoles/µg total protein in early exponential stage cells and remained almost at the same level during late exponential phase, after which it declined slightly to 66 fmols/µg total protein in the stationary phase (Figure S6B). Gre levels in exponentially growing *M. tuberculosis* cells were also comparable to the levels seen with *M. smegmatis* cells (Figure S6A). Interestingly, the combined amount of GreA (∼53 fmol/µg of total protein) and GreB (∼13 fmol/µg of total protein) [32] in exponentially growing *E. coli* cells is comparable to the level of single Gre protein found in mycobacteria. The RNAP concentration also seems to be comparable between the two species (Gupta and Nagaraja, unpublished results). Next, the expression of Gre in response to different cellular stress conditions *in M. smegmatis* was determined by measuring the protein content, and was found to be mostly unperturbed (Figure S6C). RT-PCR experiments under various conditions also did not show significant alterations in the *gre* mRNA levels (data not shown). Together, these results indicate that a constant level of Gre is retained irrespective of growth phases or environmental conditions. Above findings are in contrast to the observations in several other organisms where under different stress conditions GreA level was found to be altered [33], [34]. Thus from all the results presented in Figure 3A to 3C (*gre* knock-down) and Figure S6A to S6C, we surmise that although amount of Gre in mycobacteria is found to be comparable to *E. coli*, maintaining the level is critical for cell survival.

### Reduction of abortive transcription initiation, and anti-arrest activity of *Mtb*Gre

**Figure 4:**
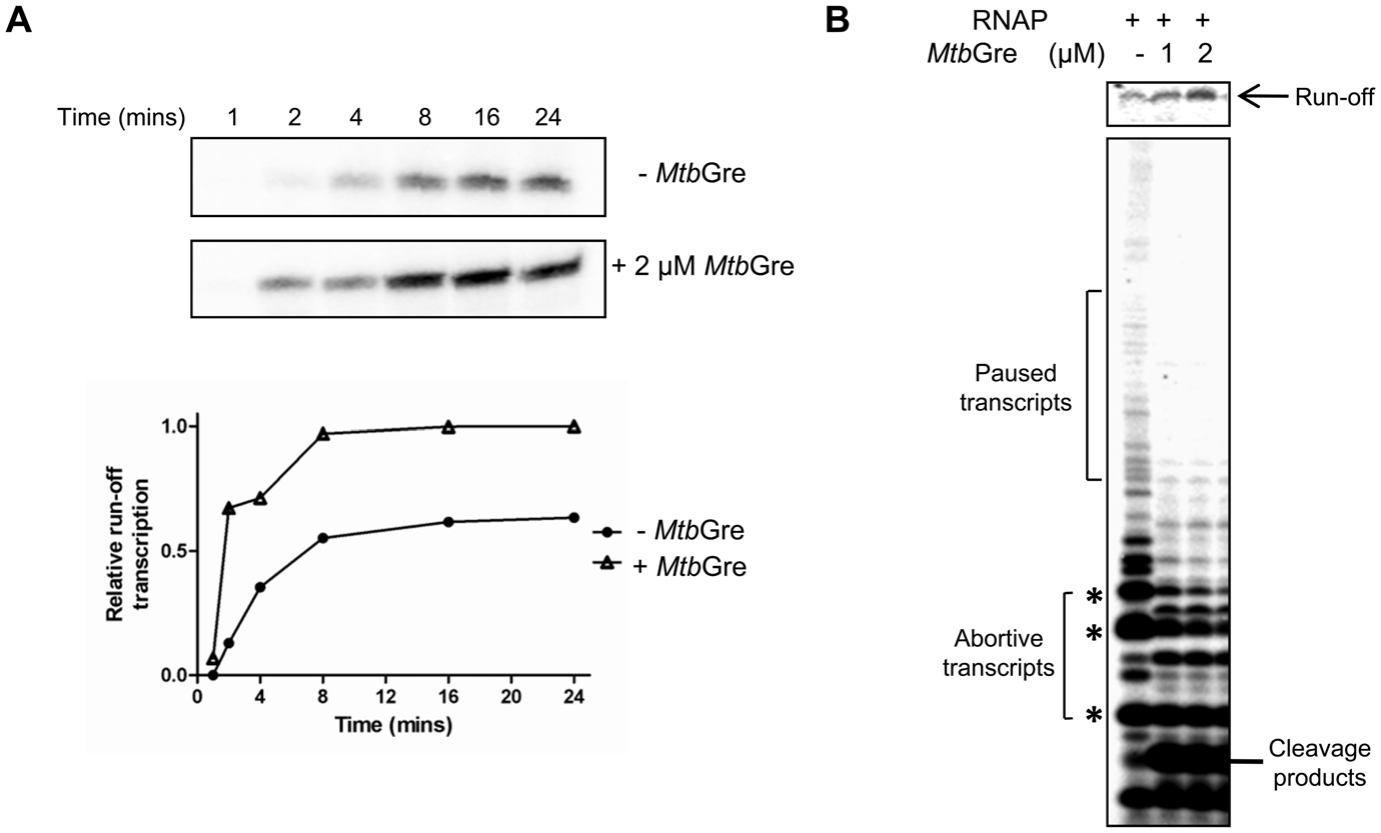
Effect of *Mtb*Gre factor on promoter clearance and abortive transcription. (**A**) Promoter clearance assays were carried out in the absence (-•-) or presence (-**Δ**-) of 2 µM *Mtb*Gre. Transcripts were resolved on an 8% urea-PAGE and 109 nt long run-off transcripts were quantified using Image Guage (Fuji Film) and plotted (lower panel). The intensity of the bands was normalized against the amount of run-off transcript produced after 24 mins in presence of *Mtb*Gre. (**B**) *Mtb*Gre reduces abortive transcript level. In this assay, transcription reactions were carried out in the absence and presence of 1 µM and 2 µM *Mtb*Gre. The reactions were resolved on a 20% urea-PAGE to visualize abortive transcripts (marked by ‘*’).

**Figure 5:**
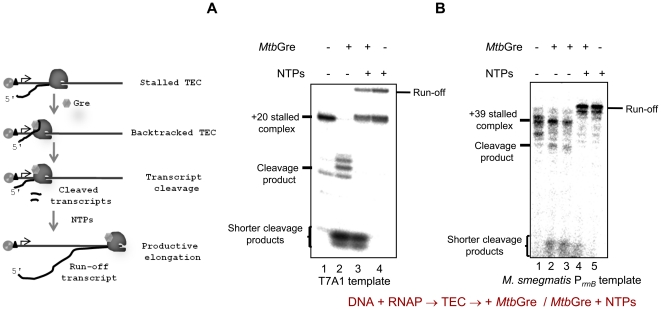
RNAP can restart transcription elongation after transcript cleavage by *Mtb*Gre. The starting materials in all the experiments were purified stalled complexes with radiolabeled RNA. (**A**) Cleavage-restart assay on T7A1-TEC. The scheme on the left depicts the reaction process. The TEC was incubated without (lane 1) or with *Mtb*Gre (lane 2 and 3), or NTPs (lane 3 and 4), as indicated. (**B**) Similar assay was carried out on *M. smegmatis* P*_rnB_* promoter template (the RNAP stalls at +39 position in the absence of UTP in the reaction mix). Transcripts were analyzed by resolving on 20% urea PAGE.

To determine the activity of *Mtb*Gre, *in vitro* transcriptions were carried out using *M. smegmatis* P*_rrnPCL1_* as a template. The efficient open complex (RP_O_) formation is not effectively transmitted to the synthesis of full length transcripts in this promoter due to high abortive RNA synthesis [35]. One of the properties of the *E. coli* Gre factors is to reduce abortive RNA synthesis and enhance promoter clearance [36], [37]. *Mtb*Gre enhanced the full-length transcript synthesis from P*_rrnPCL1_* by overcoming the abortive transcripts (Figure 4A). Notably, the intermittent pauses seen above the abortive transcripts in the transcription from P*_rrnPCL1_* were also reduced in the presence of *Mtb*Gre (Figure 4B). After the cleavage of the transcript in the paused elongation complex, the trimmed TEC was capable of restarting the transcription in presence of all NTPs from both T7A1 promoter and mycobacterial P*_rrnB_* promoter templates (Figure 5A, 5B). However, the minor differences in the patterns in Figures 5A and 5B could be template specific effect. It is possible that some of the stalled elongation complexes generated on T7A1 template have entered an inactive arrested state which could not be elongated further. Taken together, data from these experiments indicate that *Mtb*Gre factor could function on pre-formed stalled elongation complexes and induce transcript cleavage-restart activity.

### Structural features of Gre factors are conserved in *Mtb*Gre

**Figure 6:**
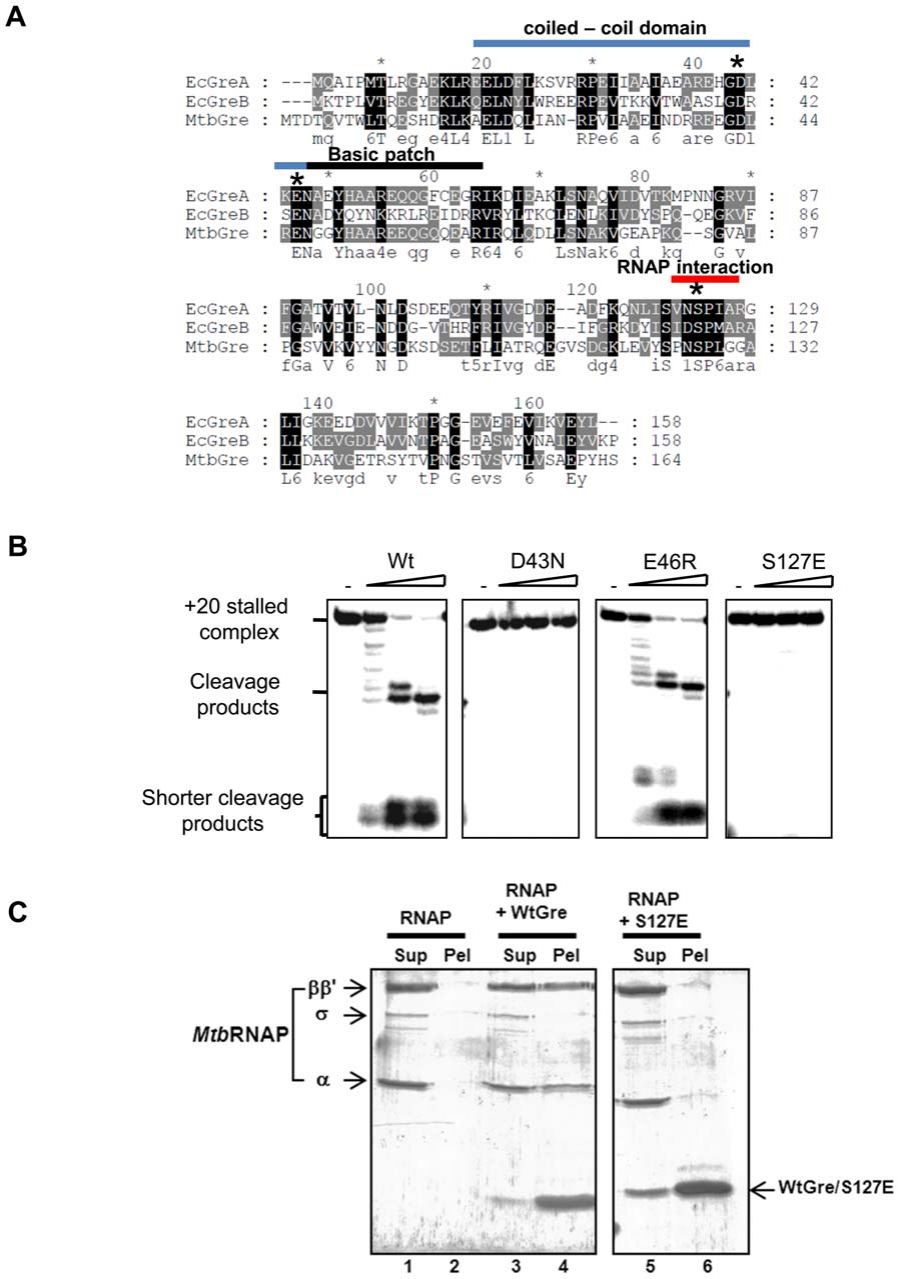
Conserved residues of *Mtb*Gre factor are important for Mg^++^ co-ordination and RNAP binding. (**A**) Multiple sequence alignment of *Mtb*Gren (164 aa) with *E. coli* GreA (158 aa) and GreB (158 aa). N-terminal coiled-coil domain is marked in blue and its basic patch in black. The C-terminus RNAP interaction domain is marked in red in the alignment. Conserved acidic residues at the tip of N- terminus coiled coil domain and S127 at the C-terminus, subjected to site directed mutagenesis are marked by an ‘*’.(**B**) Comparative activity of Wt with D43N, E46R and S127E mutants in T7A1 TEC. (**C**) Ni-NTA pull down of his-tagged Wt and S127E mutant with *Mtb*RNAP. Lane-1 and 2 represent the supernatant and pellet fraction from the control reaction having only *Mtb*RNAP. Lanes 3,4 represent the supernatant and pellet fraction of WtGre respectively and lanes 5 and 6 represents mutant S127E along with *Mtb*RNAP respectively.

Alignment of the *Mtb*Gre with its *E. coli* counterparts revealed the following conserved features (Figure 6A). (i) Acidic amino acids at the tip of the predicted coiled-coil domain found in the N-terminus of the protein. In *E. coli* Gre factors, these residues are involved in Mg^2+^ co-ordination with the RNAP active center [10]. (ii) A short basic patch of residues on one side of a helix, which interacts with the 3′ end of RNA in *E. coli* [38]. (iii) A globular domain at the C-terminus of the protein. Residues in this domain of *E. coli* GreB interact with the carboxyl-terminal coiled-coil domain of RNAP β′ subunit [27]. The D43, E46 at the acidic tip of the coiled-coil domain (equivalent to the D36 and E39 of *E. coli* GreA) and S127 at the C-terminal globular domain of *Mtb*Gre factor (equivalent of *E. coli* GreA S119) (Figure 6A) were mutated to D43N, E46R, and S127E to address their function in *Mtb*Gre. The D43N and S127E mutations completely abolished the activity of *Mtb*Gre factor. On the other hand, E46R mutant retained the cleavage stimulation activity (Figure 6B). These results indicate that among the two acidic residues in the tip of N-terminal predicted coiled-coil domain, D43 is essential for the transcript cleavage activity. The loss of activity of the S127E mutant was probably due to its loss of interaction with the RNAP. Ni-NTA pull down assays were carried out to assess the direct interaction between purified *Mtb*RNAP and histidine tagged *Mtb*Gre or its S127E variant. The *Mtb*Gre factor bound *Mtb*RNAP (Lane 4 of Figure 6C), and as predicted S127E mutant did not interact with the RNAP (Lane 6 of Figure 6C).

### *Mtb*Gre factor is specific to the mycobacterial RNAP

**Figure 7:**
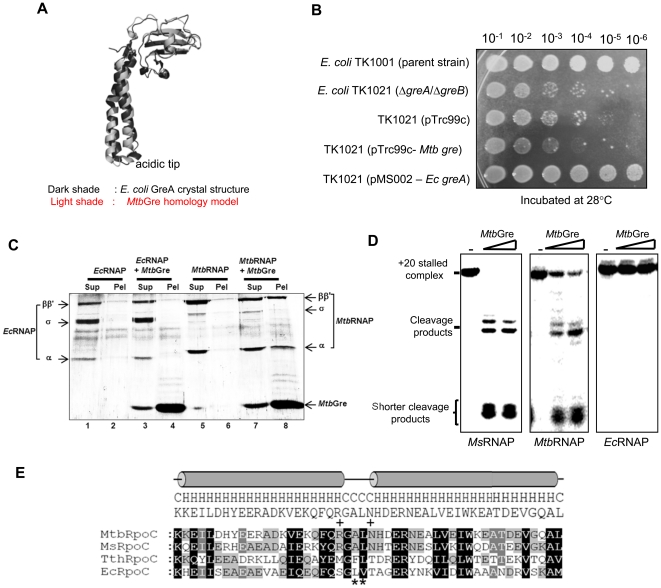
*Mtb*Gre factor is specific to mycobacterial TEC. (**A**) Homology modeling of *Mtb*Gre using *E. coli* GreA crystal structure (PDB code: 1GRJ) as template. (**B**) Complementation of *E. coli ΔgreA/ΔgreB* strain (TK1021) with *Mtb gre*. The gene was cloned into pTrc99c and the *E. coli* cells harboring different plasmids were grown till OD_600_ 0.6 and spotted onto IPTG containing plates and incubated at 28°C. (**C**) Interaction of histidine tagged *Mtb*Gre with *Ec* and *Mtb* RNAPs by Ni-NTA pull-down. Lanes1 and 2 - supernatant and pellet of only *Ec*RNAP and lanes 5 and 6 are *Mtb*RNAP from the control reactions. Lane 4 and 8 represent the pellet fractions of the reactions with *Ec* and *Mtb* RNAP with *Mtb*Gre respectively (**D**) TECs prepared with *Ms*, *Mtb* and *Ec* RNAP were incubated with *Mtb*Gre and the resulting products were resolved on a 20% urea PAGE. (**E**) Comparison of coiled-coil domain present in the C-terminus of the RNAP β′ subunit from *M. tuberculosis*, *M. smegmatis*, *T. thermophillus* and *E. coli*. Residues marked with ‘+’ are either charged or polar amino acids present in the loop region of mycobacterial β′ subunit but absent in the other two.

The *Mtb*Gre factor shares similar structural features (Figure 7A) with *E. coli* GreA and could rescue halted elongation complexes. Therefore, the ability of *Mtb*Gre to functionally complement the *E. coli* Gre factors was tested by using an *E. coli* Δ*greA*/Δ*greB* double knock-out strain [39], which shows a cold-sensitive phenotype. *Mtb*Gre factor expressed from a pTrc construct could not complement *E. coli* Δ*greA*/Δ*greB* grown at 28°C (Figure 7B) although the protein was expressed in *E. coli* (Figure S7A). The failure to complement could be due to the lack of interaction between *E. coli* RNAP and *Mtb*Gre (Figure 7C). In support of this, *in vitro* assays showed that *Mtb*Gre factor functions only on mycobacterial, i.e., *M. smegmatis* and *M. tuberculosis* TECs (Figure 7D). It did not stimulate transcript cleavage on *E. coli* RNAP containing TEC even at a very high concentration (>10 µM). Similarly, *E. coli* GreA was also not functional on the mycobacterial elongation complexes (Figure S7B).

## Discussion

In this study, we describe the characterization of *Rv1080c* - the Gre factor present in the *M. tuberculosis* genome. The *Mtb*Gre increased the transcription efficiency both during initiation and elongation phase of the process. During initiation, it reduced the abortive transcripts and enhanced the promoter clearance. At elongation phase, the protein rescued RNAP from the transcription pauses by inducing the transcript cleavage. Knocking down of the gene resulted in growth retardation and cell death indicating its essentiality for cell survival.

In organisms where Gre factors have been analyzed so far, they show remarkably similar structural features. Functional characterization of the Gre factors from *E. coli* [6], [7], *T. aquaticus* and *T. thermophilus* [15], [17], [40] revealed the conserved nature of the transcript cleavage stimulation activity required for efficient transcription process. However, *gre* genes were found to be dispensable in *E. coli*; *ΔgreA* - *ΔgreB* double knock-out strain showed only a mild cold-sensitive phenotype [39]. In contrast, in *M. tuberculosis*, the protein appears to have a more pronounced and indispensable role. In the first glance our results appear to be contradicting the earlier transposon mutagenesis studies which led to the isolation of insertional mutation of *M. tuberculosis gre* (http://mylims2.cvmbs.colostate.edu/tnlist/). We have noticed that the point of insertion of the transposon is at the 493^rd^ position out of the 495 bases in the *Rv1080c*. Thus it is likely that, the gene was not inactivated in the mutant strain. Also, with the decrease in intracellular Gre levels, the cell survival was affected. Notably, significant amount of the protein is present at all growth phases indicating its house-keeping function. Further, the Gre protein level was not altered to a great extent during different stress conditions, indicating that an optimum level of the protein may be required for cell survival.

*Mtb*Gre can rescue a pre-formed halted elongation complex to exert its anti-arrest activity similar to *E. coli* GreA and ensure efficient transcription elongation. The transcript cleavage pattern of *Mtb*Gre showed type I cleavage products i.e. predominantly 2–3 nt fragments similar to the activity of *E. coli* GreA. The longer transcript cleavage pattern (2–18 nt, type II) seen with *E. coli* GreB is mediated by a large stretch of positively charged residues in its N-terminal domain [38]. *Mtb*Gre does not have such a large stretch of basic amino acids and the surface charge distribution is similar to that of *E. coli* GreA (Figure S1C). In organisms having GreB, RNAP could backtrack farther to have a larger RNA 3′ end fragment to be processed. Indeed, in such conditions, high affinity interaction between RNAP and GreB results in transcript cleavage activity [16], [27]. Earlier studies have revealed lower transcription elongation rates in mycobacteria [41], [42]. Organisms such as *E. coli* with faster transcription rates seem to require two Gre factors to process shorter and longer RNA.

The action of the *Mtb*Gre seems to be restricted to mycobacterial transcription machinery as it did not rescue a halted elongation complex of *E. coli* RNAP. Lack of interaction between these heterologous partners could account for the observation. The interaction surface on *E. coli* RNAP for *E. coli* GreB was mapped to a conserved hydrophobic loop in the coiled-coil domain in the C-terminus of the β′ subunit [27]. The region is also conserved in the mycobacterial RNAP (Figure 7E) indicating the conserved architecture of transcription machinery. However, the C-terminal globular domain of Gre factors (GreA, GreB of *E. coli* and *Mtb*Gre), which interacts with the RNAP, shows considerable variation, although certain specific residues in the hydrophobic patch are conserved in all these proteins. Importance of specific interactions between RNAP and Gre is suggested from the studies in *T. aquaticus*. GreA of *T. aquaticus* failed to induce transcript cleavage in *Ec*RNAP elongation complexes [15] similar to the present observation with *Mtb*Gre. Thus it appears that the transcript cleavage activity requires species-specific interactions, although both partners *viz* RNAP and Gre have conserved characteristics across species. Gre may have a more important function in mycobacteria to compensate for the low intrinsic cleavage activity of mycobacterial RNAP compared to its *E. coli* and themophilic counterparts. This deficiency could affect the recovery from arrest of backtracked *Mtb*RNAP in the absence of *Mtb*Gre. The similar mechanism has been recently proposed to explain growth inhibition of the yeast strains expressing the cleavage deficient mutant of the eukaryotic Gre homolog, TFIIS [43]. The results presented here and the data emerged till date from a number of studies with Gre factors of diverse group of organisms emphasize the biological importance of these secondary channel binding proteins. The deletion of *greA* led to hypersensitivity phenotype under various stress conditions in *E. coli* [39], *Sinorhizobium meliloti* [44] and *Rhizobium tropici* [45] implicating the importance of Gre factors in the survival of the organism in the restrictive environment. In contrast, the decrease in Gre levels under normal cellular growth conditions itself reduced the viability of *M. tuberculosis*. The indispensability of the Gre factor in *M. tuberculosis* but not in *E. coli* [39] or *T. thermophilus* [17] indicates that the intracellular role of the factor is likely to be varied between different species of bacteria.

*Mtb*Gre seems to be the only transcription elongation factor in the genome possessing cleavage activity as the other ORF - Rv3788 found in the genome with lower degree of relatedness do not appear to participate in the process. The lack of transcript cleavage stimulatory activity in Rv3788 may be attributed to the absence of several key residues in the N-terminus which are found in Gre factors across different organisms. Although the two acidic residues needed for Mg^2+^ co-ordination are conserved in Rv3788 (Figure S1A), Asn47 and Tyr50 (present in *Mtb*Gre), required for binding to the backtracked protruding nascent RNA are absent. Nevertheless, Rv3788, has several features similar to the RNAP secondary channel binding proteins and hence may have some other intracellular role. It is also apparent that the RNAP secondary channel binding proteins are emerging to be the key regulators of different cellular functions apart from the transcript cleavage stimulatory functions [5].

In conclusion, Rv1080c functions like a bona fide Gre factor with transcript cleavage stimulatory activity in *M. tuberculosis*. Gre function is required for the optimal growth of the mycobacteria in contrast to its dispensability in *E. coli*. GC rich templates are known to impose blockage during transcription due to the formation of stable RNA-DNA hybrids [46]. Such strong barriers have to be overcome to ensure high fidelity RNA synthesis. Slower transcription rates in mycobacteria may lead to intermittent pauses and stalling at specific signals. Under these circumstances RNAP has to ensure completing the elongation process. Transcription factors like Gre, which maintain the efficiency by preventing premature pauses, appear to have a more profound role in maintaining the genomic integrity of *M. tuberculosis*.

## Methods

### Bacterial strains, plasmids and the growth conditions

*M. smegmatis* mc^2^155 [47] and *M. smegmatis* SM07*sigA* [48], [49] were cultured in Middlebrook 7H9 medium (Difco) containing 0.05% Tween-80 (Sigma) and 0.4% glucose (Sigma) under shaking conditions at 37°C. *M. tuberculosis* H37Ra [50] cells were cultured in Middlebrook 7H9 medium supplemented with ADC consisting of 0.2% glycerol (Sigma) and 0.05% Tween-80 at 37°C. To check the expression pattern of Gre at different growth phases in *M. smegmatis* and *M. tuberculosis*, cells were grown for 12, 18, 24, 30, 36 and 48 hrs (for *M. smegmatis*) or 3, 5, 7, 12 and 20 days (for *M. tuberculosis*), pelleted down by centrifugation, lysed by sonication and cell extracts were prepared.

Knock-down of *gre* expression in *M. smegmatis* mc^2^155 *and M. tuberculosis* H37Ra was carried out by generating the plasmid pMV*greAS* (*Mtbgre* in anti-sense orientation) in pMV261 [51]. The coding sequence was amplified using primers with BamH1 site (Table 1) and cloned downstream of the *hsp60* promoter at a BamH1 site of the vector pMV261 to generate plasmid pMV*greOE* (Table 1) for over-expression of *Mtb*Gre in both *M. smegmatis* and *M. tuberculosis*. Comparison of the growth rates of different strains was carried out by inoculating (1% inoculum) 30 ml of Middlebrook 7H9 medium with 25 µg ml^−1^ kanamycin to obtain an initial OD_600_ of 0.02 to 0.04. Growth of the strains was monitored by dilution - plating from 8 day culture of *M. tuberculosis* or 20 hrs cultures of *M. smegmatis* grown at 37°C in shaking conditions. The cells were diluted in fresh media and plated into the middlebrook 7H10 agar plates to determine the cell viability by counting the cfu.

**Table 1:** Oligonucleotides, strains and plasmids used in this study.

Name	Description	Reference
*M. smegmatis* SM07*sigA*	(Hyg^R^, his-*rpoC*, pJAM2*mysA*)	[46], [47]
*M. smegmatis mc^2^* 155	(A high efficiency transformation strain of *M. smegmatis*)	[45]
*M. tuberculosis H37Ra*	(An attenuated strain of *M. tuberculosis* H37Ra)	[48]
*E. coli* BL21	(*hsdS gal (λcIts857 ind1 Sam7 nin5 lacUV5-T7 gene 1*)	[51]
*E. coli* TK1001	MC1061 *zgj-203*::Tn*10*	[39]
*E. coli* TK1021	MC1061 *greA*::*kan*, *greB*::*cat*, *zgj-203*::Tn*10*	[39]
pMS002	Derivative of pBR322 containing *Mtb greA* gene	[39]
pMV261	*E. coli*-mycobacteria shuttle vector with a *hsp60* promoter	[49]
pET20b*gre*	*M. tuberculosis gre* cloned between NdeI and HincII of pET20b	This study
pET20b*rv3788*	*M. tuberculosis Rv3788* cloned between NdeI and HindIII of pET20b	This study
pET20b*msgre*	*M. smegmatis gre* cloned between NdeI and HindIII of pET20b	This study
pET20b*gre*-his	*M. tuberculosis gre* cloned in pET20bwith C-terminus his-tag	This study
pET20b*gre*-his	*M. tuberculosis gre* S127E cloned in pET20b with C-terminus his-tag	This study
pET20b*EcgreA*-his	*E. coli greA* cloned in pET20b with C-terminus his-tag	This study
pMV*greAS*	*M. tuberculosis gre* cloned in anti-sense orientation under *hsp60* promoter	This study
pMV*greOE*	*M. tuberculosis gre* coading sequence cloned under *hsp60* promoter	This study
pTrc99*gre*	*M. tuberculosis gre* cloned under *trc* promoter	This study
Gre D43N Mut	5′ GAAGAAGGCAACCTGCGCGAGAAC 3′	This study
Gre E46R Mut	5′ GAAGGCGACCTGCGCCGTAACGGCGGATACCAC 3′	This study
Gre S127E Mut	5′ TACTCGCCGAATGAACCGCTCGGTGGG 3′	This study
greBamH1For	5′ ACGGATCCCGACCATATGACGGATACTCAAGTC 3′	This study
greBamH1Rev	5′ ACGGATCCCGACCTGCTCGGAGATCTCGAACAG 3′	This study
greNdeIFor	5′ CGACCATATGACGGATACTCAAGTC 3′	This study
greHindIIIRev	5′ ATAAGCTTCGACCTGCTCGGAGATCTCGAACAG 3′	This study
rv3788NdeIFor	5′ ATGCGACATATGAGCGAGAAAGTCGAGTC 3′	This study
rv3788HindIIIRev	5′ ATAAGCTTTTCTGAGGGCAGCTTGACAG 3′	This study
MsgreNdeIFor	5′ ATGCGACATATGACCGATACCCAGGTCACC 3′	This study
MsgreHindIIIRev	5′ ATAAGCTTTCCGCCTTGATACGGCTCAGC 3′	This study

### Western blots

To detect the protein level at different growth phases, cell lysates were probed for Gre factor with a polyclonal antibody raised in mice and anti-SigA antibody in rabbit. The primary antibodies were probed with the secondary antibody coupled with HRP and blots were developed using a chemiluminescence substrate (GE Health Care). Expression of Gre factor during different stress conditions were also checked by growing *M. smegmatis* cells till mid-log phase and subjecting them to varied stresses as described [52]. The amount of Gre protein present in the *M. smegmatis* cells was determined by western blot. Varying concentrations of the purified *M. smegmatis* Gre were loaded in the same gel as standards along with 120 µg of cell extracts from different growth phase cultures and subsequently probed with anti-Gre antibody.

### Microscopy

*M. smegmatis* cells harboring pMV261 or pMV*greAS* or pMV*greOE* constructs were grown in Middlebrook 7H9 medium at 37°C to mid-exponential phase. Cells were pre-fixed in PBS, 1% (v/v) Triton X-100 (Sigma) and 2% (v/v) toluene (Merck) solution and incubated overnight at 4°C. Cells were stained with DAPI solution (4′,6-diamidino-2-phenylindole), which binds specifically to DNA. Microscopic observations were carried out by using a Carl Zeiss fluorescent microscope at 1000× magnification.

### Expression and purification of *Mtb*Gre, *Ms*Gre and *Rv3788*

*gre* (*Rv1080c*) and *Rv3788* genes were PCR amplified from *M. tuberculosis* genomic DNA with specific primers (Table 1) and cloned between the NdeI and HindIII site of pET20b (pET20b*gre* and pET20b*rv3788*). The *M. smegmatis gre* (*MSMEG_5263*) gene was PCR amplified from *M. smegmatis mc^2^155* genomic DNA and cloned in pET20b (between NdeI and HindIII site). Site directed mutants of *Mtbgre* were generated using the mega-primer inverse PCR method with pET20b*gre* as a template (primer sequences are listed in Table 1). The purification of *Mtb*Gre, its mutants and *Ms*Gre was carried out as follows. *E. coli* BL21 cells [53] with pET20b*gre* or its mutants or pET20b*msgre* were grown till OD_600_ 0.6 at 37°C and induced with 0.3 mM IPTG. Cells were lysed by sonication and centrifuged at 100,000 g for 2 hrs. The supernatants were subjected to 0–65% ammonium sulfate precipitation and re-suspended in 3 ml of TGE buffer [10 mM Tris-HCl, pH 8.0, 5% glycerol, 0.1 mM EDTA] with 50 mM NaCl and subsequently resolved by a 120 ml Sephacryl S-100 gel filtration column. The fractions having Gre protein were further purified through DEAE - Sephacel chromatography by eluting with a linear NaCl gradient of 50 mM to 400 mM. The Rv3788 protein was purified from the *E. coli* BL21 cells harboring pET20b*rv3788*. The purification involved a 45–60% ammonium sulfate precipitation of the cell lysate followed by DEAE - Sephacel chromatography. All the proteins purified were approximately 95% pure as judged by SDS-PAGE (Figure S2C). From 2 liters each of the cultures overexpressing the proteins (*Mtb*Gre, *Ms*Gre and Rv3788), about 5 mg of each of the protein were obtained. *E. coli greA* was cloned with a C-terminal His-tag in pET20b and the protein was purified from *E. coli* BL21 cells [53] over-expressing the protein using a Ni-NTA column. *M. smegmatis* RNAP was purified by following the method described earlier [49]. *M. tuberculosis* RNAP was purified from 2 liters of *M. tuberculosis* H37Ra cells grown for 8 days at 37°C in MB7H9 medium with ADC supplement (Difco). The purification involved gel filtration on Superdex S-200 matrix and subsequent heparin - Sepharose chromatography following the method described for native *M. smegmatis* RNAP purification [49].

### Promoter clearance and abortive transcription

100 nM of RNAP and 20 nM of *M. smegmatis* P*_rrnPCL1_* promoter containing template were incubated in transcription buffer [50 mM Tris-HCl pH 7.5, 10 mM MgCl_2_, 100 µM DTT, 5% glycerol, 50 µg ml^−1^ BSA and 100 mM KCl] for 15 mins at 37°C to form the open complex. Subsequently 50 µg ml^−1^ heparin was added to the reactions and incubated for 1 min. Transcription was initiated by the addition of 100 µM NTPs and 2 µCi of α-^32^P[UTP]. Aliquots were withdrawn at different time intervals and reactions were incubated for indicated time. Reactions were analyzed in 22% urea-PAGE to resolve the abortive products.

### Stalled TEC preparation

Transcription assays were carried out using T7A1 promoter and RNAPs from *E. coli*, *M. smegmatis* and *M. tuberculosis*. Ternary elongation complexes were generated on a 5′ biotinylated T7A1 promoter-containing DNA template (Figure S3A). The TECs for *E. coli* or the mycobacterial RNAPs were prepared by following the methods described for *E. coli* and *T. thermophillus* enzymes [15], [26]. RP_O_ were formed by incubating 100 nM of *M. smegmatis*, *M. tuberculosis* or *E. coli* RNAP and 15 nM T7A1 promoter containing template DNA at 37°C for 15 min in transcription buffer. For multiple round transcription assays, 100 µM of NTPs were added to the reaction mix and incubated further for 15 mins at 37°C. Reactions were stopped with formamide dye and analyzed in Urea PAGE. For stalled complex formation assays, after RP_O_ formation, 100 µM ATP, 100 µM GTP and 2 µCi [α-^32^P] ATP (300 Ci mmol^−1^, Perkin Elmer) were added. Reactions were carried out in the absence of CTP and UTP to generate stalled elongation complexes containing a 20 mer transcript. *M. smegmatis* P*_rrnB_* promoter template was used for the preparation of mycobacterial TEC. RNAP stalls at +39 position in the absence of UTP in this template. TECs were further purified by mixing 5 µl of Streptavidin-Sepharose beads (GE Healthcare) to each reaction and precipitated by centrifugation. Pellets containing the elongation complexes were washed thrice with transcription buffer supplemented with 200 mM KCl and 100 µg ml^−1^ heparin followed by washing twice with only transcription buffer. Indicated amounts of *Mtb*Gre or Rv3788 were added to the beads re-suspended in the transcription buffer followed by incubation at 37°C for 30 min. 150 nM *Mtb*Gre was found to be optimum for cleavage of 50% of the T7A1 TECs and was thus defined as the unit activity. Reactions were terminated with the addition of formamide dye [0.025% (w/v) bromophenol blue, 0.025% (w/v) xylene cyanol FF, 0.08% amaranth (w/v), 10 mM EDTA, 0.025% SDS and 80% deionized formamide] and RNA cleavage products were analyzed by electrophoresis in a 20% denaturing PAGE. Amaranth dye included in the formamide stop mix served as a size marker. In a 22% urea PAGE the dye moves at a position corresponding to 2–3 nt short RNA fragments [54].

### Intrinsic cleavage activity of RNAP

Intrinsic cleavage activity of the *M. smegmatis*, *M. tuberculosis* and *E. coli* RNAPs was detected by prolonged incubation (up to 4 hrs) of the TECs (prepared with 15 nM template and 100 nM RNAP) in transcription buffer (pH 7.5) at 37°C followed by resolving in a 20% urea - acrylamide gel. pH - induced transcript cleavage reactions were carried out in three different buffer systems at 37°C for 30 mins. (i) 40 mM PIPES adjusted to pH 6.0 by addition of 1 M NaOH; (ii) 40 mM Tris adjusted to pH 7.0, 8.0, and 9.0 by the addition of 1 M HCI; (iii) 40 mM CAPS adjusted to pH 10.0 by the addition of 1 M NaOH. All buffers contained 0.1 M KCl and 10 mM MgCl_2_.

### Cleavage-restart activity of *Mtb*Gre

The 20 mer T7A1 TEC or the 39 mer *M. smegmatis* P*_rrnB_* TECs were prepared by using 15 nM biotinylated template and 100 nM RNAP. The RNAP was stalled at T7A1 template by using only 100 µM of ATP, GTP and 2 µCi of [α-^32^P] ATP (300 Ci mmol^−1^, Perkin Elmer) in each of the 10 µl reaction volume. For generating +39 stalled elongation complex at P*_rrnB_* promoter, 100 µM of ATP, GTP and CTP were used along with 2 µCi of [α-^32^P] ATP. To detect cleavage-restart activity of *Mtb*Gre, the TECs were incubated with the *Mtb*Gre factor in presence or absence all the four NTPs. Initially the complexes were incubated with 2 µM of *Mtb*Gre for 30 min followed by the addition of the NTPs and incubation was continued for another 10 min followed by resolving in a 20% urea PAGE.

### *Mtb*Gre-RNAP interaction

C-terminal his-tagged *Mt*bGre and its S127E mutant were cloned in pET20b and purified using a Ni-NTA column. 5 µg of both RNAP (*Ec* or *Mtb*) and Gre protein were used for analyzing direct interactions. Proteins were incubated together for 15 mins in 50 µl volume of incubation buffer containing 50 mM tris - HCl (pH 8.0), 100 mM potassium glutamate, 5% glycerol, and 20 mM imidazole at room temperature. 20 µl of Ni-NTA pre-equilibrated with incubation buffer was then added to the protein mixture and incubated for an additional 30 mins in a rotary mixer. The supernatant was separated and the pellet was washed thrice with 400 µl of the incubation buffer. Finally, the pellet was re-suspended in 50 µl of buffer mixed with SDS-gel loading buffer, boiled and loaded onto an 11% SDS-PAGE along with the supernatant fractions followed by silver staining of the gel.

### Complementation of *E. coli ΔgreA*/*ΔgreB* strain with *M. tuberculosis* gre

The *M. tuberculosis gre* gene was cloned in pTrc99c vector to obtain pTrc99*gre* construct which was used for complementing the *E. coli* TK1021 strain (Table 1). The parental strain TK1001 was used as wild type *E. coli* control. *E. coli greA* expressing plasmid pMS002 was used as a positive control in these experiments [39]. The cells were grown in liquid culture and different dilutions were spotted on LB plates containing 0.3 mM IPTG and appropriate antibiotics (Table 1).

## Supporting Information

Figure S1 **Sequence alignments and homology modeling of Gre.** (**A**) Multiple sequence alignment of the *E. coli* GreA and GreB with *Mtb*Gre and the Rv3788 using ClustalW program (http://www.ebi.ac.uk/Tools/msa/clustalw2). The alignment figure is created using GenDoc – multiple sequence alignment editor (http://www.psc.edu/biomed/genedoc). The conserved amino acids are shaded in black and substitutions with similar amino acids in grey. The conserved acidic residues at the N-terminus are labeled as “♦” and hydrophobic residues in the C-terminus as “•”. (**B**) Homology model of the *Mtb*Gre and Rv3788 (using *E. coli* GreA crystal structure – 1GRJ as template). Models were generated using the comparative protein structure modeling program Modeller ver. 9.3. [Eswar, N et al. Comparative Protein Structure Modeling With MODELLER. Current Protocols in Bioinformatics, John Wiley & Sons, Inc., Supplement 15, 5.6.1–5.6.30, 2006]. (**C**) Surface charge distribution of *Mtb*Gre and Rv3788. Positively charged surface is shown in blue and the negatively charged region in red. *E. coli* GreA structure (Stebbins et al. [23]) is shown on left and GreB (Vassylyeva et al. [27]) second from the left and compared with the *Mtb*Gre (second from right) and Rv3788 (rightmost). Positively charged region on the surface of the coiled-coil domain is shown in the box.
(TIF)

Figure S2 **Over-expression of Gre and Rv3788.** (**A**) Gre factors of both *M. tuberculosis* and *M. smegmatis* were over-expressed and purified from *E. coli* BL21 cells. UN: un induced cell lysates and IN: induced cell lysate of *Mtb*Gre and *Ms*Gre over-expressing cells respectively. (**B**) Over-expression of *M. tuberculosis* Rv3788 in *E. coli* BL21 cells. Both un induced and IPTG induced samples of Rv3788 expressing cells show robust hyper-expression. (**C**) Purified proteins: *Mtb*Gre (17.8 kDa), Rv3788 (17.4 kDa) and *Ms*Gre (18 kDa). The yield of all the three proteins was ∼5 mg from 2 liters of culture. The proteins were >95% pure.
(TIF)

Figure S3 **Transcription assays with T7A1 promoter templates using mycobacterial RNAPs.** (**A**) A modified T7A1 promoter was used for generating the stalled complexes. Residues underlined are the ones replaced from the original residues showed above. Biotin tag is present at the 5′ end of the template. In presence of ATP and GTP, RNAP forms a stalled TEC with a 20mer RNA. (**B**) Multiple-round *in vitro* transcription with *E. coli*, *M. smegmatis* and *M. tuberculosis* RNAP from T7A1 promoter. Abortive transcripts are indicated in the lower panel.
(TIF)

Figure S4 ***Ms*** **and** ***Mtb*** **RNAP are free from endogenous Gre factor contamination.** 20 µg of both *Ms* and *Mtb* RNAP were probed with anti-Gre antibody. 100 ng of purified *Mtb*Gre was used as a control.
(TIF)

Figure S5 **Activity of** ***Ms*** **Gre and the phenotypic effects of** ***gre*** **overexpression.** (**A**) Transcript cleavage stimulatory activity of *Ms*Gre. (**B**) SDS-PAGE analysis of the cell lysates from *M. smegmatis* with pMV261, with the over-expression construct (pMV*greOE*) and antisense (pMV*greAS*) mediated knock-down construct. (**C**) Morphology of the *M. smegmatis* cells over-expressing *Mtb*Gre. Comparison of cellular morphology (left panel) and nucleoid (right panel) of *M. smegmatis* mc^2^155 cells harboring either the pMV261 vector or pMV-*greAS* or pMV-*greOE* constructs. Left panels- bright-field images; right panels-fluorescent images showing the DAPI-stained nucleoid.
(TIF)

Figure S6 **Determination of the expression pattern of Gre and its amount in the cells.** (**A**) Expression of *gre* in response to different stresses in *M. smegmatis* and *M. tuberculosis* determined by western blot. (**B**) Estimation of the level of Gre protein in the *M. smegmatis* cells. Purified *M. smegmatis* Gre protein was used as a standard. 120 µg of total cell lysate proteins were probed with the anti-Gre antibody to estimate the Gre protein level in cells at different growth phases. (**C**) Western blot analysis for Gre from cell lysate of *M. smegmatis* exposed to different stresses.
(TIF)

Figure S7 **Expression of Gre from pTrc** ***gre*** **construct in** ***E. coli*** **TK1021.** (**A**) The gel shows expression of *Mtb*Gre from the pTrc*gre* construct, induced with 0.3 mM IPTG. (**B**) Transcript cleavage assays using *Mtb*Gre and *E. coli* GreA. Only *Mtb*Gre shows cleavage of mycobacterial RNAP elongation complex.
(TIF)
