# Supplementary figures and images for: A Transcript Cleavage Factor of Mycobacterium tuberculosis Important for Its Survival
Source: PLoS One. 2011 Jul 8;6(7):e21941. doi: 10.1371/journal.pone.0021941 (PMC3132773; doi:10.1371/journal.pone.0021941)

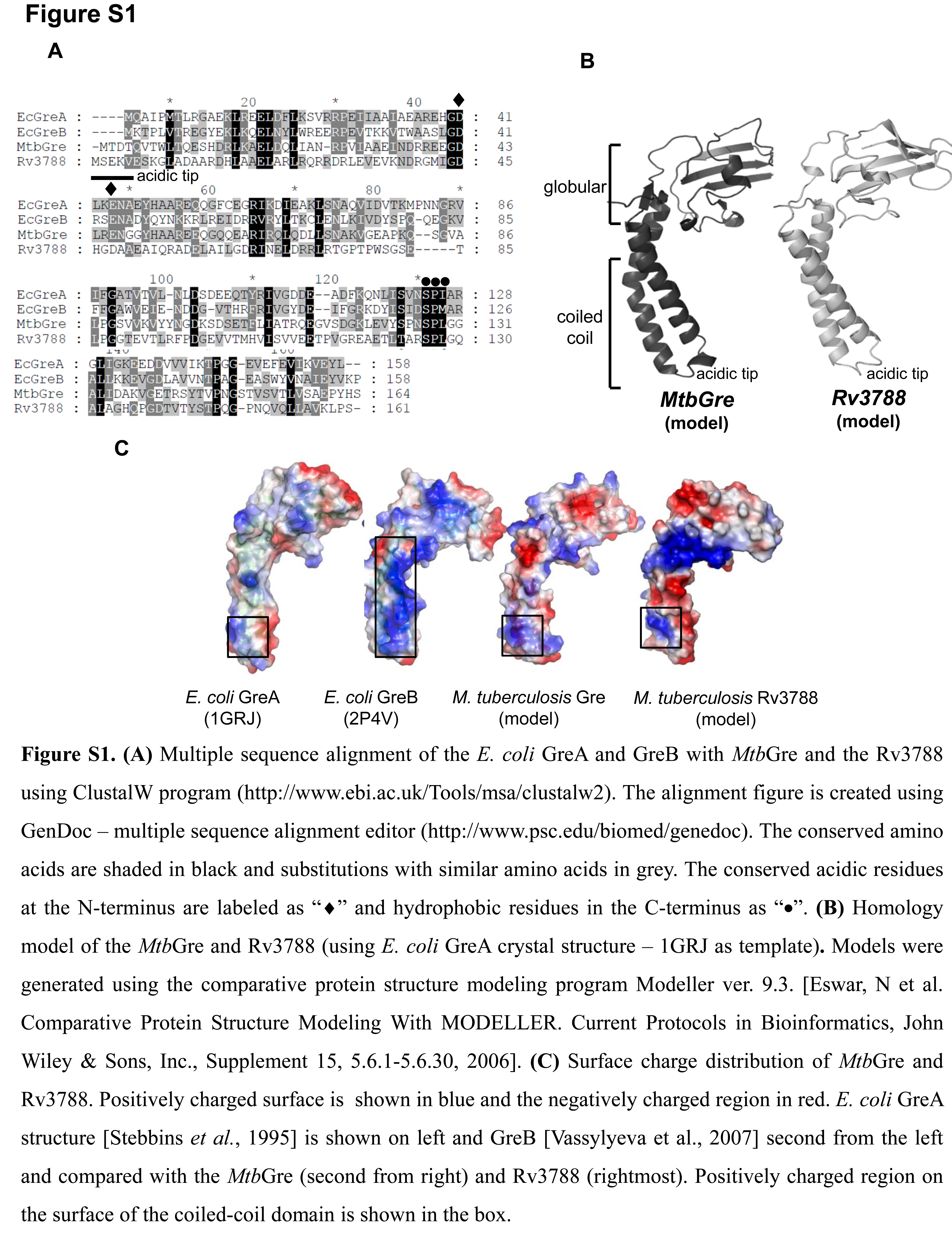

Supplement: Figure S1 — Sequence alignments and homology modeling of Gre. (A) Multiple sequence alignment of the E. coli GreA and GreB with MtbGre and the Rv3788 using ClustalW program (http://www.ebi.ac.uk/Tools/msa/clustalw2). The alignment figure is created using GenDoc – multiple sequence alignment editor (http://www.psc.edu/biomed/genedoc). The conserved amino acids are shaded in black and substitutions with similar amino acids in grey. The conserved acidic residues at the N-terminus are labeled as “♦” and hydrophobic residues in the C-terminus as “•”. (B) Homology model of the MtbGre and Rv3788 (using E. coli GreA crystal structure – 1GRJ as template). Models were generated using the comparative protein structure modeling program Modeller ver. 9.3. [Eswar, N et al. Comparative Protein Structure Modeling With MODELLER. Current Protocols in Bioinformatics, John Wiley & Sons, Inc., Supplement 15, 5.6.1–5.6.30, 2006]. (C) Surface charge distribution of MtbGre and Rv3788. Positively charged surface is shown in blue and the negatively charged region in red. E. coli GreA structure (Stebbins et al. [23]) is shown on left and GreB (Vassylyeva et al. [27]) second from the left and compared with the MtbGre (second from right) and Rv3788 (rightmost). Positively charged region on the surface of the coiled-coil domain is shown in the box. (TIF) [file pone.0021941.s001.tif]

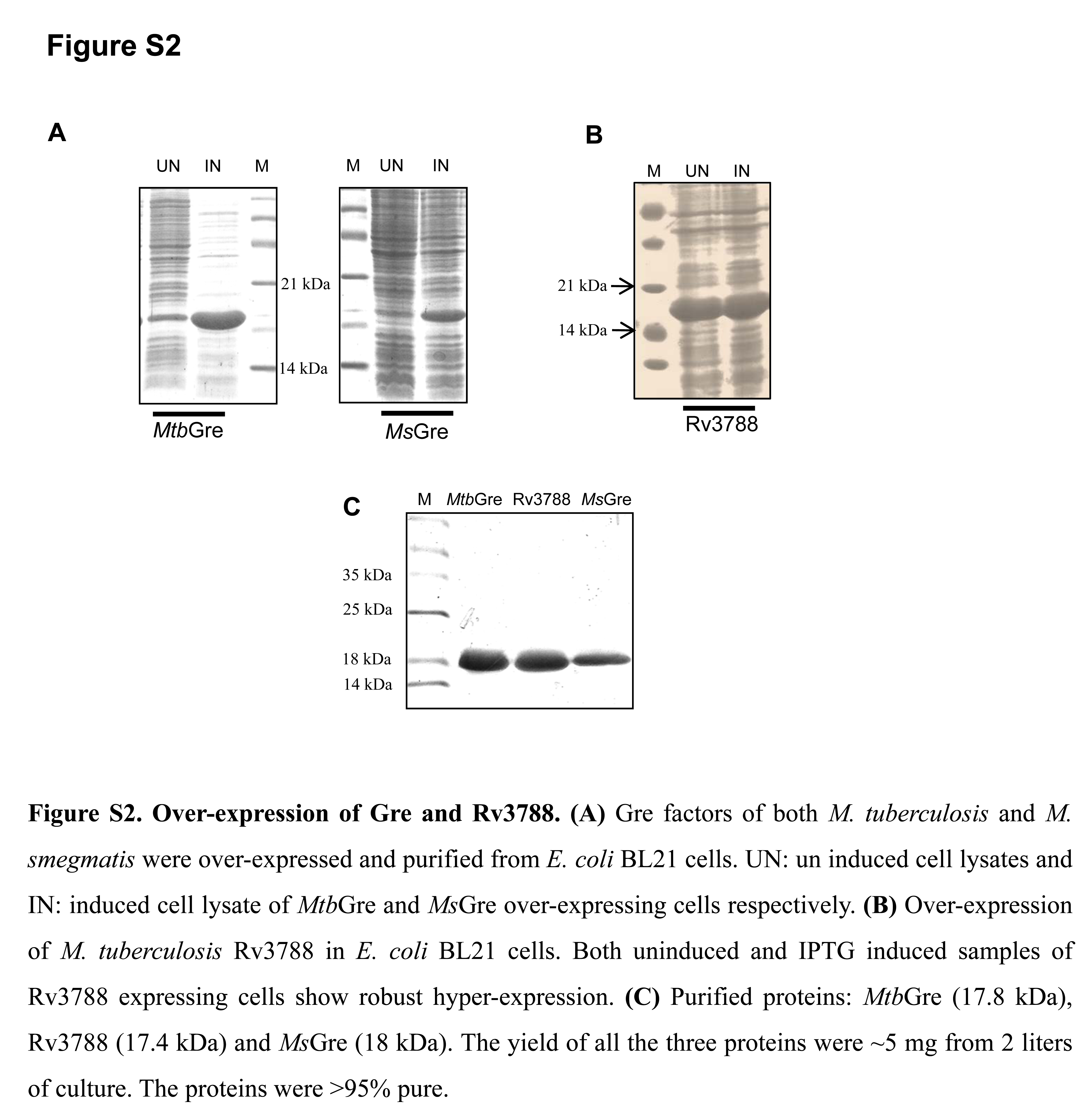

Supplement: Figure S2 — Over-expression of Gre and Rv3788. (A) Gre factors of both M. tuberculosis and M. smegmatis were over-expressed and purified from E. coli BL21 cells. UN: un induced cell lysates and IN: induced cell lysate of MtbGre and MsGre over-expressing cells respectively. (B) Over-expression of M. tuberculosis Rv3788 in E. coli BL21 cells. Both un induced and IPTG induced samples of Rv3788 expressing cells show robust hyper-expression. (C) Purified proteins: MtbGre (17.8 kDa), Rv3788 (17.4 kDa) and MsGre (18 kDa). The yield of all the three proteins was ∼5 mg from 2 liters of culture. The proteins were >95% pure. (TIF) [file pone.0021941.s002.tif]

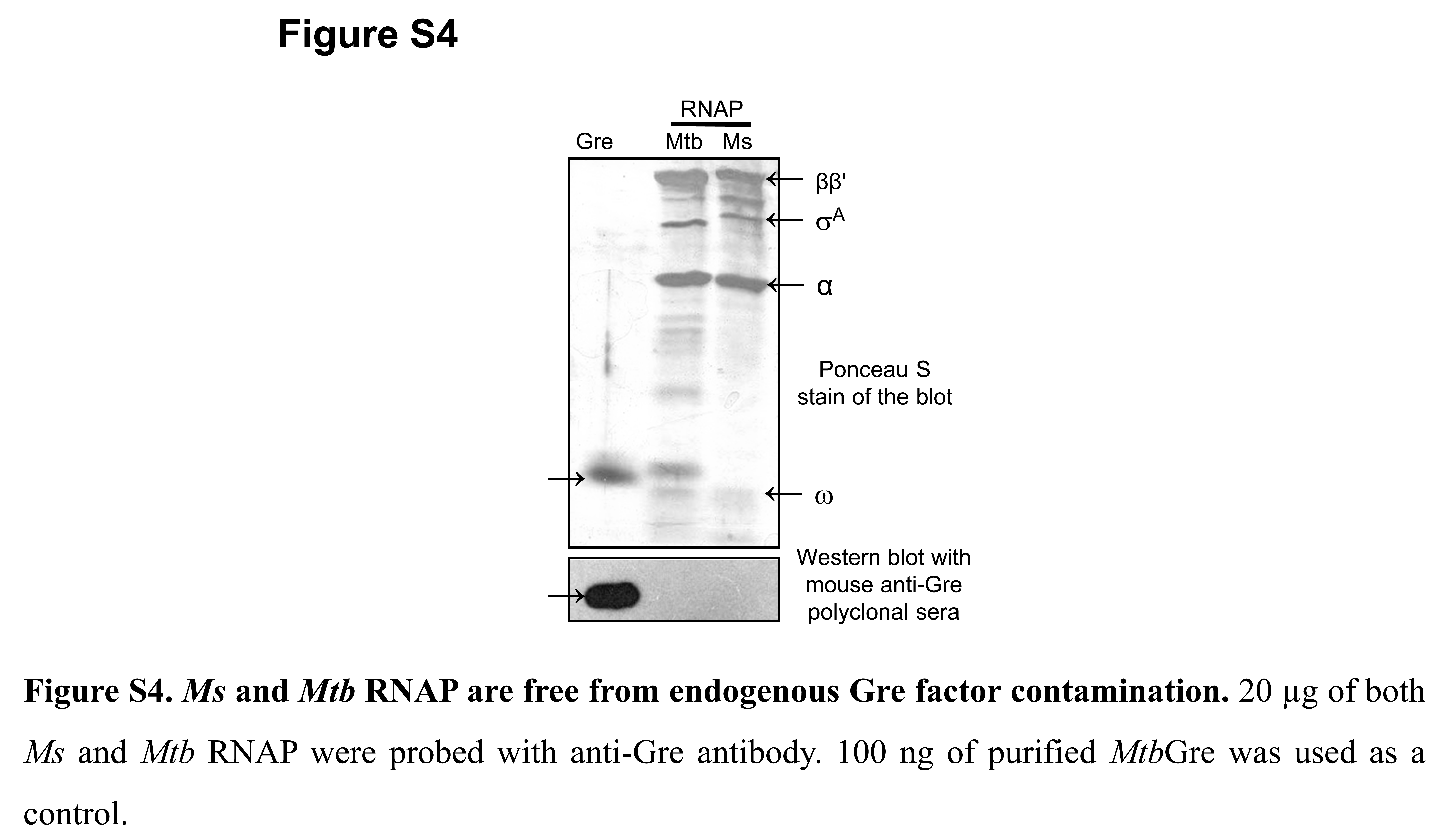

Supplement: Figure S4 — Ms and Mtb RNAP are free from endogenous Gre factor contamination. 20 µg of both Ms and Mtb RNAP were probed with anti-Gre antibody. 100 ng of purified MtbGre was used as a control. (TIF) [file pone.0021941.s004.tif]
